# “*I’m Not Only a Body*”: Change in Thoughts about the Body after Mirror Exposure Treatment in Women with Obesity—An Exploratory Study

**DOI:** 10.3390/healthcare12060624

**Published:** 2024-03-09

**Authors:** Cristina González-Sánchez, José Jiménez-Cabello, Sonia Rodríguez-Ruíz, José Luis Mata-Martín

**Affiliations:** 1 Department of Personality, Evaluation and Psychological Treatment, University of Granada, 18012 Granada, Spain; cgsanchez@ugr.es (C.G.-S.); srruiz@ugr.es (S.R.-R.); matamar@ugr.es (J.L.M.-M.); 2 Department of Sociology, University of Granada, 18001 Granada, Spain

**Keywords:** body dissatisfaction, body discomfort, exposure treatment, obesity, qualitative

## Abstract

Nowadays, obesity (OB) is one of the most important health problems in population-wide health. In addition to its physical consequences, it is a risk factor for the development of psychological problems, including body dissatisfaction (BD). This is why the treatment of BD is essential for its prevention. However, this has mostly been studied from a quantitative perspective, without focusing on the discomfort experienced by the person and the accompanying thoughts and emotions. In this study, 26 women with obesity (BMI > 30 kg/m^2^) participated, of whom 16 had high BD and 10 had low BD, as measured by the BSQ questionnaire. The women with high BD underwent six sessions of exposure to their own body in front of a mirror, recording the discomfort experienced with this vision during the session. In addition, all participants recorded positive and negative thoughts towards their body before and after these sessions. After the exposure treatment sessions, a reduction in symptomatology (BD, discomfort when visualizing one’s own body) was observed, as well as a change in the thoughts expressed by the participants, both in quantity (fewer negative thoughts) and in quality (a more positive self-perception and/or in more respectful terms used towards themselves). In conclusion, such treatments prove to be effective in reducing subjective discomfort and body-related thoughts in women with obesity.

## 1. Introduction

Obesity is defined as an abnormal or excessive accumulation of energy in the form of body fat, due to a number of complex and multifactorial causes [1], which ultimately has health, social and economic consequences [2,3]. Overweight and obesity are defined by the WHO [4] as having a body mass index (BMI) equal to or greater than 25 kg/m^2^ and 30 kg/m^2^, respectively. 

The prevalence of obesity (OB) has been increasing considerably in both adult and child populations, especially in industrialized countries [3]. According to the 2020 European Health Survey in Spain, 16% of the population over 18 years of age is obese (15.5% women and 16.5% men) and 37.5% of people (30.6% women and 44.9% men) are overweight. This increase in the prevalence of obesity and overweight in the population, together with their serious health consequences, means that this epidemic is considered a public health problem [5,6].

Obesity, however, is not only characterized at a physical level, but can also be considered both a cause and an effect of alterations at a psychological level, including negative thoughts or emotions (e.g., worry or sadness) [7], changes in certain personality characteristics (e.g., reduced behavioral control) [8], and changes in various psychopathologies associated with weight itself (e.g., alterations in eating behavior or preoccupation with one’s figure) [9].

There are a number of psychological variables that play a role in the development of body image problems and eating disorders, including low self-esteem, perfectionism, social (or media) pressure, social (or peer) comparison, and the existence of and exposure to a constant thin ideal [10,11]. 

One of the models that has best explained body dissatisfaction, involving most of those variables that can have an effect on body image and eating disorders, is the so-called “Tripartite Influence Model” [12]. This model states that social ideals of beauty are established in society, transmitted, and reinforced by three main sociocultural factors: the peer group, the family, and the media. In this way, the model maintains that these three influences contribute to social comparison based on appearance and the internalization of the thin ideal, which in turn results in this body dissatisfaction [12,13]. Thus, the model really suggests that body dissatisfaction (BD) is a result of the influence of psychological processes related to self-esteem and all the external influences related to appearance to which the person is exposed in their daily life [14]. Therefore, the basis of BD is formed of negative thoughts about one’s own appearance, for example, the belief that other people do not like their body or that they cannot show it freely for fear of being judged by their environment [14].

With all this, we can understand that BD becomes, therefore, one of the fundamental factors in the development, maintenance, and relapse of eating disorders (EDs) [15,16]. In addition, it can lead to what are known as body image disturbances, which are defined as a complex [17].

Multiple studies have demonstrated the relationship between BMI and BD, such that the higher the BMI, the greater the person’s body dissatisfaction [18]. Therefore, people with obesity (or overweight) will be at greater risk of experiencing high dissatisfaction compared to those with normal weight [19,20]. However, studies investigating the diagnosis, evaluation, and treatment of BD mostly tend to focus on people with EDs [15] and hardly on obesity [21]. As for the diagnosis of BD in EDs and OB [22], although this has not yet been consciously studied, there is great variability in present incidence rates [23]. On the other hand, epidemiological studies are scarce and usually use screening instruments to determine the risk of disease, i.e., questionnaires such as the Body Shape Questionnaire (BSQ [24]) or the Eating Disorders Inventory (EDI [25]); alternatively, they are based on the diagnostic criteria described in the Diagnostic and Statistical Manual of Mental Disorders (DSM-5 [26]).

More studies have tried to investigate the effectiveness of different treatments in reducing BD and the maintenance of those results in the long term. Thus, most research supports the use of treatments that include exposure to the body through a mirror, this being a fundamental element of body image therapy [19,20]. Self-exposure to the body has been shown to be effective in reducing BD [27] alongside the treatment of the negative emotions and feelings present derived from this exposure in people with obesity [28] or EDs [19].

Among body exposure treatments, the two most widely used and verified modalities are guided exposure (the person is told which parts of the body to pay attention to: “beautiful” or “ugly” parts) and pure exposure (the person must pay attention to their whole body, with no guidance as to which parts to attend to). The literature finds conflicting results on whether guided exposure treatments show a greater reduction in BD, especially focusing on body parts considered “negative/ugly” [19,20] compared to those without such guidance [16,29,30]. However, one fact that has been widely established is that both can be clinically effective in improving BD, body checking/avoidance behaviors, and patients’ moods [16,31].

Previous research showing the effectiveness of exposure therapy for patients with body dissatisfaction generally measures its effectiveness at a subjective level by means of questionnaires [18] or by recording the discomfort experienced by individuals during treatment [32]. When other types of measures have been used, unfortunately, either no conclusive results have been obtained [33] or measures were only recorded in one treatment session, thereby not obtaining real results regarding the effectiveness of the treatment [34].

In addition, negative thoughts towards body image and body shape occur as a unique and conclusive factor in the origin of this BD and the symptomatology that may be associated with it [14,35]. It is known that externalizing thoughts about the body appears to help decrease BD and ED symptomatology [36], so studying the thoughts of people with obesity during treatment using a qualitative methodology could be key to understanding, from a more global perspective, the progress made during the sessions. However, there are few studies that use this type of methodology, and even fewer longitudinal studies.

Due to the wide controversy found in the literature and the aforementioned studies, the present study was designed with two objectives in mind: firstly, to analyze the possible changes that could be produced in the levels of body dissatisfaction and discomfort within the body in women with obesity after receiving pure mirror exposure treatment, and secondly, to observe the modification after the treatment of the thoughts directed towards the body, the extent of these thoughts, and the content of these thoughts.

## 2. Materials and Methods

### 2.1. Participants

The final sample consists of twenty-six university women with OB who voluntarily participated in this study. The initial sample was 1878 women, with the final participants selected according to their BMI (BMI > 30 kg/m^2^) and the score obtained in the *Body Shape Questionnaire* (BSQ [24]), thus forming the two comparison groups in the present study.

**Figure 1:**
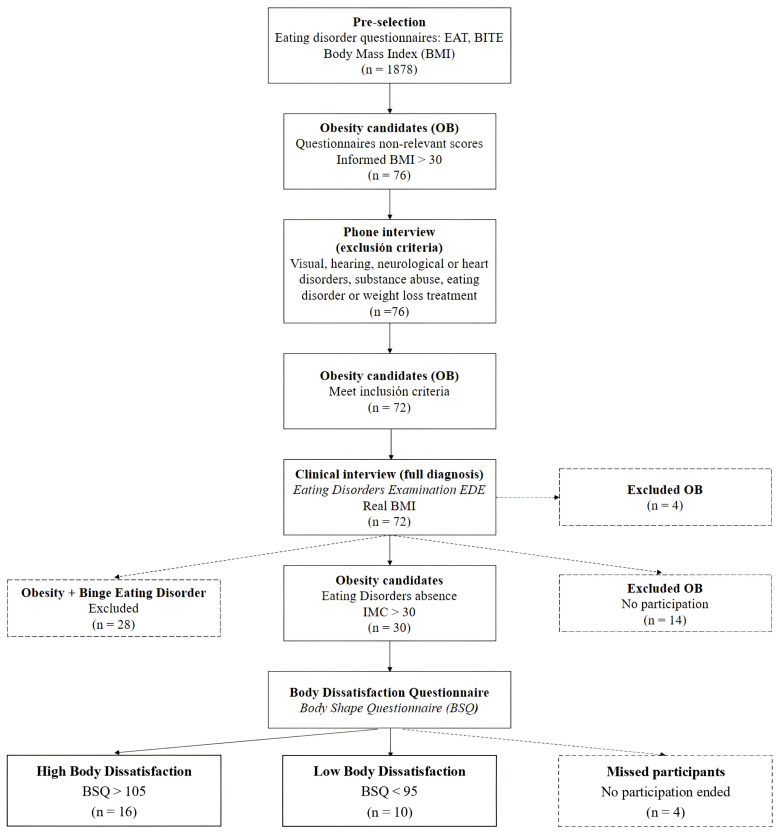
Stages and selection process of the final sample.

Once the requirements for experimental participation were verified, 76 women were contacted and a telephone interview was carried out to verify that they did not meet any of our exclusion criteria: (1) existence of purgative behaviors and/or binge eating, (2) receiving treatment for weight loss, (3) frequently consuming alcohol and/or drugs, (4) receiving psychological and/or pharmacological treatment of some type, and (5) having previously undergone some body image treatment. Afterwards, the sample was reduced to a total of 72 candidates. After a first clinical interview (Eating Disorder Examination, EDE), 14 participants were unwilling and/or unable to participate in the experiment or to participate in the study and receive the treatment sessions; 28 women were diagnosed with binge eating disorder criteria. Finally, the BSQ questionnaire was provided to the remaining 30 participants, forming a final sample of 16 women with high body dissatisfaction (BSQ > 105) and 10 women with low body dissatisfaction (BSQ < 90) for the present study. The remaining four participants did not complete the treatment sessions. These processes can be observed in Figure 1. 

### 2.2. Procedure

The 26 participants who agreed to participate in the study signed an informed consent form and were weighed and measured in the laboratory with a body composition scale (Tanita Model 300MA, Chicago, IL, USA), ensuring they met obesity criteria (BMI > 30 kg/m^2^) and had not suffered weight changes. 

On the one hand, participants with high BD received 6 individual treatment sessions lasting approximately 45 min 2 times per week, with a total duration of 3 weeks. The time of the treatment session remained at the time chosen by each participant. Participants were financially compensated for their participation. On the other hand, the participants in the control group did not receive such sessions and were scheduled to complete the questionnaires after 3 weeks, coinciding with the time of the study in both groups. 

After the end of each of the treatment sessions, participants completed the following questionnaires: the Body Appreciation Scale (BAS [37], available in Supplementary Materials) and the Visual Analogue Scale (VAS), together with the recording of positive and negative thoughts about their own body. In addition, after the end of the treatment sessions, the Body Shape Questionnaire (BSQ [24]) was provided again, in order to measure changes in body dissatisfaction in all participants.

### 2.3. Treatment

The treatment consisted of 6 sessions of pure exposure in front of the mirror. these sessions lasted approximately 45 min and were received at a rate of 2 sessions per week. During the treatment sessions (carried out by an expert body image psychologist), participants were asked to observe their body in front of a winged mirror, which allowed them to see themselves from all angles, including the rear view of the body. In each of these sessions, they were invited to attend to and accept those thoughts and feelings that might arise, without offering resistance to them, while continuing to attentively observe their body. In order to reduce exposure avoidance, participants could follow a completely free path of their body, indicating which points they were focusing their attention on. Simultaneously with the exposure, they reflected the thoughts that were emerging towards their body, as well as associated sensations and emotions, in addition to the level of discomfort they experienced during the different moments of the session. 

### 2.4. Measures

*Body Shape Questionnaire* [BSQ] [24]. The BSQ (available in Supplementary Materials) consists of 34 items assessing IC, fear of weight gain, concern about appearance, and extreme desires to lose weight. In the present study, was used the Spanish adaptation and validation of the BSQ [38] (see Supplementary Materials).

*Visual Analogue Scale* [VAS]. The VAS is a visual scale for the assessment of dissatisfaction/satisfaction and ugliness/beauty. The scale is answered by marking the point at which the individual being assessed would be considered to be on a continuum across a 100 mm long line, such that the score is considered to be from 0 (at the negative end) to 100 (at the positive end).

*Body Appreciation Scale* [BAS] [37]. The BAS is a scale for the evaluation of the acceptance and care of the individual towards his or her own body. For this research, we used the version adapted and validated in Spanish [39].

*Recording of positive and negative thoughts.* We used a recording template for recording the number (up to a maximum of 5) and frequency of occurrence (from 1 to 5) of positive and negative thoughts about each person’s body, as well as the annotation of the thought itself.

### 2.5. Analysis

Between-group differences in questionnaire scores (means and SD in: BAS, VAS–satisfaction and VAS–beauty) before and after the treatment sessions were analyzed using univariate ANOVAs, as well as controlling for other study variables (e.g., BMI). The results obtained between sessions for the number and frequency of occurrence of the thoughts of the participants in the experimental group have been analyzed by means of a 2 (thoughts: positive and negative) × 6 (session: 1–6) ANOVA. Analyses of the scores obtained by participants in the experimental group within and between treatment sessions on the VAS (Satisfaction and Beauty) scales were performed using an ANOVA 2 (phase: pre- and post-session) × 6 (Session: 1–6). All statistical analyzes were performed using SPSS v25. Greenhouse–Geisser correction was used, reporting original degrees of freedom and corrected *p* values. Multiple comparison analyzes were performed using the Bonferroni test. A value of 0.05 was set to establish statistical significance. The figures included in the article were obtained using Graphpad Prism 9.0.2, for Windows (GraphPad Software, Boston, MA, USA).

On the other hand, the content analysis technique was used; this is a methodology applied in the field of social sciences that allows the grouping of thematic categories [40]. The selection of this technique is justified by the fact that this research aims to go a step further by delving into the meanings and experiences of the participants in this study [41]. It should be noted that within the different existing forms for the collection of qualitative information, the strategy of open answers in questionnaires was used [42].

The implementation of this process, from the beginning until the results are obtained, follows the scheme proposed by Mayring [43]: (1) delimitation of the units of analysis; (2) mapping of the data; (3) the use of coding tools; (4) development of categories; (5) review of the categories; and (6) presentation of the results. The computer software ATLAS.ti version 23 was used to carry out the analysis.

## 3. Results

### 3.1. Negative and Positive Thoughts

**Figure 2:**
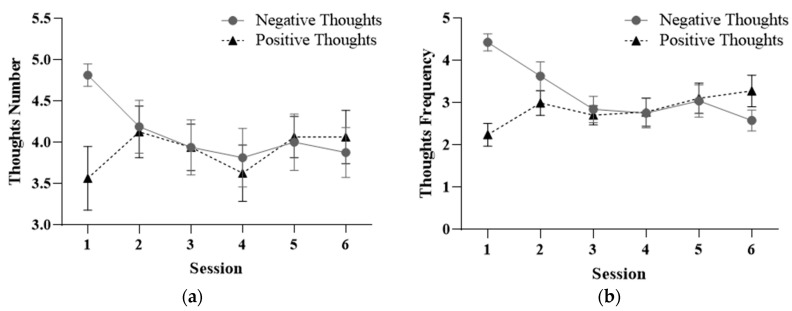
Mean scores and standard deviation of the number (**a**) and frequency (**b**) of positive and negative thoughts between treatment sessions.

The results of the repeated-measures ANOVA 2 (*Thoughts*: positive and negative) × 6 (*Session*: 1–6) were assessed using the thought log. In the case of the analysis of the number of these thoughts, the results show statistically significant differences in the intersection *Session* × *Thoughts* (F_5,75_ = 4.627; *p =* 0.008). The remaining factors and interactions do not show statistical significance. The results can be seen in Figure 2.

Post hoc results (after performing a repeated-measures ANOVAs) found statistically significant differences when comparing the number of negative thoughts (F_5,75_ = 3.042; *p* = 0.034) between sessions. As Figure 2 shows, the number of negative thoughts about the body decreased across sessions. This decrease shows a linear trend (F_1,15_ = 10.082; *p* = 0.006). On the other hand, the increase in the number of positive thoughts about the body, although there were no statistically significant differences over the sessions (*p* = 0.308), shows a cubic trend in its progress (F_1,15_ = 4.582; *p* = 0.049).

Regarding the frequency of positive and negative thoughts across sessions, the results have been analyzed using a repeated-measures ANOVA 2 (*Thoughts*: positive and negative) × 6 (*Session*: 1–6). The results obtained show statistically significant differences in the *Session* factor (F_5,75_ = 2.601; *p* = 0.032) and in the interaction *Session* × *Thoughts* (F_5,75_ = 12.377; *p* = 0.000).

Post hoc analysis using repeated-measures ANOVAs shows statistically significant differences when comparing both the frequency of occurrence of negative (F_5,75_= 9.416; *p* = 0.000) and positive (F_5,75_= 3.579; *p* = 0.027) thoughts between sessions. As shown in Figure 2B, the frequency of the occurrence of negative thoughts decreased, while the frequency of the occurrence of positive thoughts increased.

No statistically significant differences were found in the *Thoughts* factor (F_9,135_ = 0.578; *p* = 0.589). However, the post hoc results obtained (through a *t*-test analysis for independent samples) show statistically significant differences between sessions 1 and 6 (*p* < 0.011). This indicates changes in the number and frequency of thoughts about the body in the expected direction. 

### 3.2. Body Satisfaction and Perceptions

**Figure 3:**
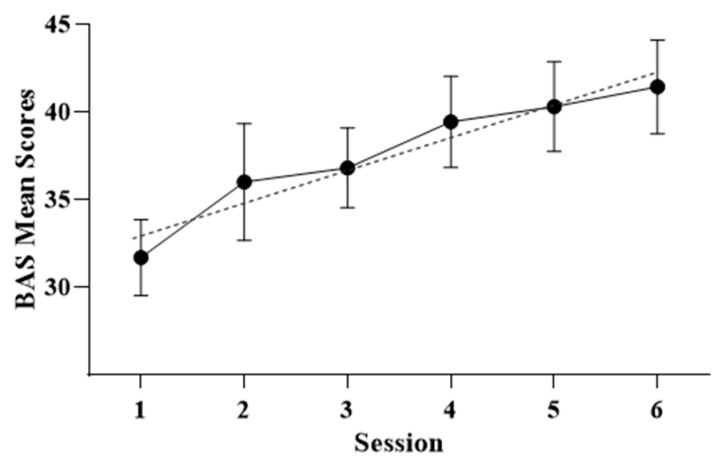
Mean scores, standard deviation, and trend in BAS questionnaire scores after exposure treatment sessions.

After performing a repeated-measures ANOVA of the BAS questionnaire from six treatment sessions, statistically significant differences were observed (F_5,75_ = 7.077; *p* = 0.001). The results can be seen in Figure 3.

As can be seen, body satisfaction increased over the sessions. This increase shows a linear trend (F_1,15_= 17.759; *p* = 0.001). This result translates into an increase in body satisfaction expressed by the participants over the treatment sessions. 

**Figure 4:**
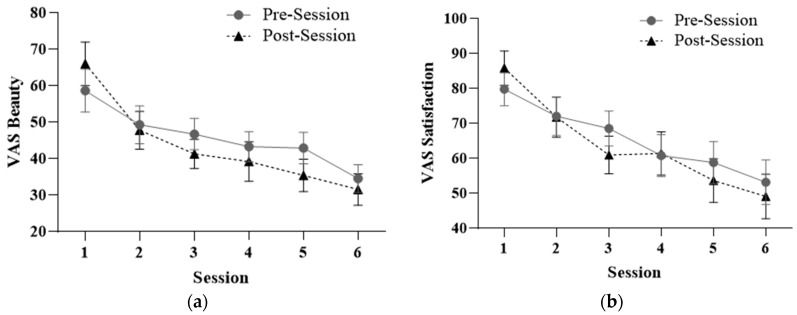
Mean and standard deviation in VAS–satisfaction (**a**) and VAS–beauty (**b**) within and between treatment sessions.

The results of the 2 (*Phase*: pre- and post-treatment) × 6 (*Session*: 1–6) repeated-measures ANOVA for the VAS–satisfaction measure show significant effects in the *Session* factor (F_5,75_ = 16.866; *p* = 0.000) and in the *Session* × *Phase* interaction (F_5,75_ = 2.588; *p* = 0.033). In the analysis of the VAS–beauty measure, significant effects are shown in the *Session* factor (F_5,75_ = 10.794; *p* = 0.000) and in the *Session* × *Phase* interaction (F_5,75_ = 2.700; *p* = 0.027). The results can be seen in Figure 4.

The significant effect of the *Session* × *Phase* interaction in both logs indicates a decrease in body dissatisfaction within the treatment session itself. 

Post hoc analyses using a repeated-measures ANOVA for changes between treatment sessions at both baseline and endline show statistically significant differences in the VAS scores recorded (*p* < 0.010). Thus, treatment effects translated into therapeutic improvements both within and between sessions.

### 3.3. Treatment Results

The results of the one-way ANOVA (*Phase*: pre- and post-treatment) for the questionnaires used in the experimental group reveal statistically significant differences after treatment in all measures except for the BSQ questionnaire, although there was a decrease in the score obtained. On the other hand, no changes were observed in the control group wherein no intervention was carried out. The results are shown in Table 1.

The changes obtained in the scores of the participants who received treatment demonstrate an improvement of symptoms after treatment. In relation to this, the content of the emotions/feelings and symptoms expressed by the participants in the study are discussed in more detail below. For this purpose, the results are presented according to types of thinking (negative or positive) and group (experimental or control).

### 3.4. About Thoughts

#### 3.4.1. Negative Thoughts in High Body Dissatisfaction 

**Figure 5:**
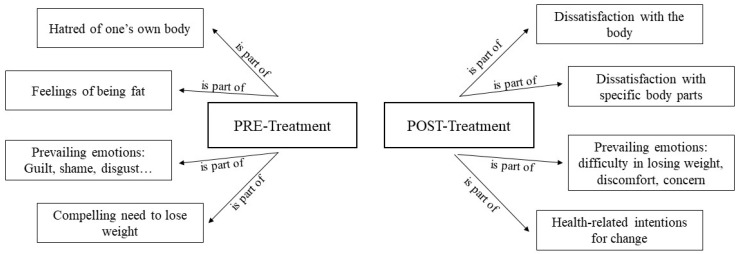
Negative thoughts in high body dissatisfaction.

The results found after the application of the content analysis technique allow us to observe how the events and feelings largely experienced in the before treatment revolved around aspects such as hatred towards one’s own body and a self-perception of looking/being fat (Figure 5). In addition, feelings of guilt and shame emerged alongside a desire to lose weight. 

However, focusing on the categories related to negative thoughts after treatment sessions (*Session* 6: post-treatment), a decrease in these feelings was observed. In this sense, reference was made to a dissatisfaction with certain parts of the body and even dissatisfaction with the body, but no such feelings of hatred, guilt, or shame appeared. Finally, the desire to lose weight was no longer mentioned as much as the existence of a less pressing need.

#### 3.4.2. Positive Thoughts in High Body Dissatisfaction

**Figure 6:**
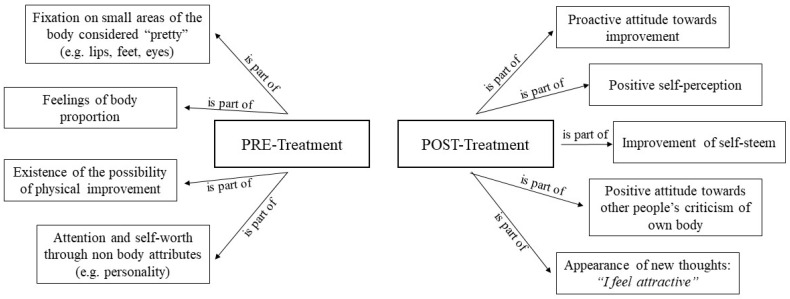
Positive thoughts in high body dissatisfaction.

From the pre-treatment phase (Session 1), up to four main categories can be highlighted: areas of the body considered beautiful, feeling proportionate, the possibility of improvement, and attention to physical attributes not related to weight. In the post-treatment part, feelings/emotions and symptoms of an even more positive nature were observed. Thus, the desire to improve, feeling attractive, feeling unaffected by criticism, self-esteem, and positive self-perception became relevant. In general, it can be noted that the positive aspects emerged more at the end of these sessions (Session 6), as can be seen in Figure 6.

#### 3.4.3. Negative Thoughts in Low Body Dissatisfaction (Control Group) 

**Figure 7:**
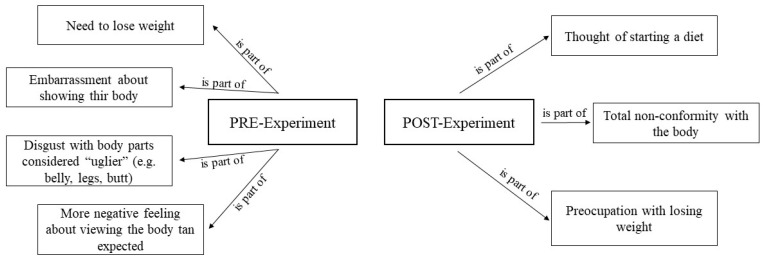
Negative thoughts in low body dissatisfaction.

In this group, it can be observed that the negative thoughts in the previous phase (before experimental sessions; this *group does not take part in exposure treatment sessions*) revolved around the need to lose weight, a more negative feeling about themselves than they had initially thought, and a feeling of hatred towards certain parts of the body. This feeling is not the only one that emerged, as linked to it is the shame of showing one’s own body (Figure 7). 

These feelings/emotions change after experimental sessions; they are not as negative as in the previous phase and focus on aspects such as the possibility of dieting, concern about losing weight, and the existence of discomfort with certain parts of the body.

#### 3.4.4. Positive Thoughts in Low Body Dissatisfaction (Control Group) 

**Figure 8:**
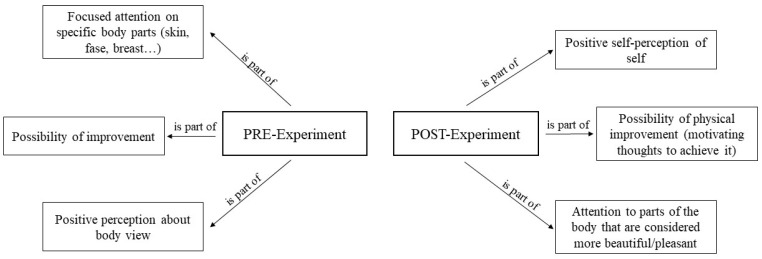
Positive thoughts in low body dissatisfaction.

Finally, it can be seen that in the first session, positive aspects were shown, such as a more positive perception than previously thought, the pointing out of beautiful parts of the body, and the possibility of improvement. In accordance with this, in the last session, this positive self-perception was pointed out again, in a more categorical way. Certain parts of the body were pointed out again, and reference was made to the individuals’ capacity for improvement (see Figure 8).

All in all, we can observe concordance in the quantitative results obtained: there was a change in the emotions/feelings and symptoms declared by the people participating in the study that seemed to lead to a reduction in what can be considered negative aspects.

## 4. Discussion

This study aimed to measure changes in body dissatisfaction in women with obesity after receiving a pure mirror exposure treatment in terms of discomfort with the visualization of their own body before, during, and after the sessions. In conjunction with this, we sought to assess whether these changes are accompanied by changes in the type of thoughts reported by the participants, in terms of quantity (number of negative thoughts) and quality (direction of those thoughts).

From the results obtained, we can observe that the treatment generated various positive effects according to the therapeutic objectives, such as a decrease in negative emotions and thoughts alongside an increase in positive feelings and body satisfaction, as has been demonstrated in previous research [31,32,44]. 

The increase in body satisfaction can be observed throughout the entire therapeutic process, which could be perceived as a process of habituation to the vision of one’s own body in the mirror. That is, this increase occurs progressively and follows a linear pattern. These results confirm those found in the literature both in the normal population [45] and in those with Eds [31,32,46] and obesity [20]. From a theoretical perspective, these results suggest that the mechanisms involved correspond to the inhibitory learning model [47], since the fear that may exist at the beginning of the treatment sessions does not really determine the therapeutic change, as stated classical habituation models [48]. This fact may also be indicative of the need to increase the number of exposure sessions so that habituation occurs, resulting in greater maintenance of results and improvement of symptoms.

Regarding the thoughts reported by the participants towards their own body, on the one hand, a decrease in the number of negative thoughts in favor of positive ones was observed. The fact of being able to verbalize a greater number of positive affirmations towards one’s own body helps to avoid focusing attention on body imperfections, which, in turn, contributes to a decrease in body dissatisfaction [49]. Moreover, the fact that the frequency of the thoughts goes hand in hand with this strengthens this hypothesis that the treatment is not only effective in reducing body dissatisfaction in terms of reduced discomfort but that the resulting shift in attentional bias [16,47]—encouraging (not implicitly) observation of more pleasant body parts—contributes to greater therapeutic relevance and effectiveness.

In addition, the content of these thoughts themselves, in the case of participants with high BD, became more benevolent, i.e., the criticisms they made of their own bodies were more constructive and less harsh towards themselves, so that the feelings of guilt and negative emotions that might be associated with them decreased, giving way to a kinder tone (e.g., changing from “*I hate my body*” to “*I think I could improve*”) and resulting more often in phrases that, although still negative, were not so absolutist and harmful towards the self. Positive thoughts, on the other hand, in addition to the notable increase in their number that occurred over the course of the treatment sessions, changed in content, becoming more general (e.g., from focusing on specific features, such as the eyes or mouth, to beginning to focus on the body in general). Being able to generate such positive thoughts towards an aversive stimulus such as one’s own body is a key step in body exposure therapy, as explained by Tanck [50].

The fact that positive thoughts increased to the detriment of negative thoughts suggests that the treatment had an effect not only on reducing body dissatisfaction per se but also on the participants’ ability to focus their attention on parts of the body that previously either went unnoticed; some participants also became able to consider something they liked about their own body when they previously had not been able to [44,51].

In the case of participants without BD, these changes in the recording of positive and negative thoughts, although they may exist, were not clinically relevant and may be due to having paid more attention to the body during the time between sessions; thus, the results are attributable to the therapeutic intervention.

## 5. Limitations and Future Research

To our knowledge, this is the first study that analyzes, jointly, from a quantitative and qualitative perspective, the changes produced in the thoughts, emotions and discomfort felt by women with body dissatisfaction when visualizing their bodies before, during, and after the therapy. 

Its limitations point to several future research directions. Firstly, the sample size of this study was small; this can be attributed to the demanding inclusion and exclusion criteria in addition to the study’s duration, making participation difficult. Second, the laboratory setting may have influenced the answers given by the participants. However, this effect decreased as the number of sessions increased. Research is needed to determine whether exposure to the body in multiple contexts will enhance the generalization of new learning and thus lead to a greater reduction in body discomfort and the negative thoughts associated with it [52]. Thirdly, the body itself constitutes a great source of stimuli, making the extinction process associated with exposure therapies difficult. Therefore, the symptoms of these disorders are extremely difficult to extinguish because they involve various cognitive, emotional, attentional, and even behavioral responses [53]. Finally, although the use of the content analysis technique fulfilled the function of delving into the meanings, experiences, and thoughts of the participants in this study, it did not allow us to delve into the discourses of the people. For this reason, as a future line of research, it would be interesting to establish a qualitative research design, using an interview technique, which will allow for a deeper understanding of the phenomenon analyzed and a better understanding of this social reality [54]. In addition to this, we will consider future research conducted to show whether women may also feel more motivated to change their lifestyle after this type of treatment and whether they achieve weight loss and weight maintenance in the long term.

## 6. Conclusions

Our results suggest that exposure treatment involving viewing the body in front of a mirror, together with verbalization of the thoughts and feelings generated by this viewing, has effects on patients’ self-evaluation of their own body. These changes are reflected in the number of negative thoughts they may have towards their body and its shape. In other words, patients become more self-indulgent and are able to think in more positive terms more frequently, and this leads to a greater acceptance of their own body and a significant reduction in the discomfort experienced. This leads to a more positive perception of the self, generating greater self-esteem, which undoubtedly has an impact on the well-being of the person and their psychological health, and this may even help to change their habits and maintenance of a healthy body. 

For these reasons, future research should further investigate BD, evaluating it with standardized and validated questionnaires as well as qualitative methods [54]. In fact, the latter can help to better understand the personal meaning that people with OB infer from their bodily discomfort, thus helping the professional and healthcare team involved in the treatment of these patients to better understand those aspects, which can then be investigated and evaluated thoroughly [55]. Finally, in our opinion, these and other interventions aimed at treating obesity (from a multidisciplinary perspective) will help to increase the success rate of such intervention programs.

## Figures and Tables

**Table 1:** Scores in pre- and post-treatment questionnaires and statistical significance in the respective T-tests in the different groups.

	Control Group				Experimental Group			
Clinical Measures	Pre-Treatment	Post-Treatment	T	*p* Value	Pre-Treatment	Post-Treatment	T	*p* Value
BSQ	69.60 (17.27)	73.40 (25.36)	−0.742	0.477	147.06 (25.88)	120.19 (40.19)	2.440	0.280
BAS	50.00 (7.51)	50.00 (7.51)	0.445	0.668	32.38 (8.93)	42.19 (13.56)	2.996	0.010 *
VAS–beauty	22.40 (15.52)	27.11 (16.09)	−0.492	0.636	64.37 (25.88)	28.50 (17.91)	5.075	0.000 ***
VAS–satisfaction	18.10 (15.84)	33.22 (15.77)	−2.507	0.037 *	73.93 (24.27)	47.00 (26.45)	2.77	0.000 ***

Notes: mean and standard deviation (in parentheses). BSQ (Body Shape Questionnaire), BAS (Body Appreciation Scale), VAS (Visual Analogue Scale). * *p* < 0.05, *** *p* < 0.001.

## Data Availability

Data are contained within the article and Supplementary Materials.

## Supplementary Materials

The following supporting information can be downloaded at: https://www.mdpi.com/article/10.3390/healthcare12060624/s1, Supplementary S1. Body Shape Questionnaire items. Supplementary S2. Body Appreciation Scale items.
