# Supplementary material for: “I’m Not Only a Body”: Change in Thoughts about the Body after Mirror Exposure Treatment in Women with Obesity—An Exploratory Study
Source: Healthcare (Basel). 2024 Mar 9;12(6):624. doi: 10.3390/healthcare12060624 (PMC10970317; doi:10.3390/healthcare12060624)
Supplement: Supplementary file 1 [file healthcare-12-00624-s001.zip › healthcare-2866154-supplementary.pdf]

Supplementary Materials

Supplementary S1. Body Shape Questionnaire items

1. Has feeling bored made you brood about your shape?

2. Have you been so worried about your shape that you have been feeling you ought to diet?

3. Have you thought that your thighs, hips, or bottom are too large for the rest of you?

4. Have you been worried that you might become fat (or fatter)?

5. Have you been worried about your flesh not being firm enough?

6. Has feeling full (e.g., after eating a large meal) made you feel fat?

7. Have you felt so bad about your shape that you have cried?

8. Have you avoided running because your flesh might wobble?

9. Has being with thin women made you self-conscious about your shape?

10. Have you worried about your thighs spreading out when sitting down?

11. Has eating even a small amount of food made you feel fat?

12. Have you noticed the shape of other women and felt that your own shape compared unfavorably?

13. Has thinking about your shape interfered with your ability to concentrate (e.g., while watching television, reading, listening to conversations)?

14. Has being naked, such as when taking a bath, made you feel fat?

15. Have you avoided wearing clothes which make you particularly aware of the shape of your body?

16. Have you imagined cutting off fleshy areas of your body?

17. Has eating sweets, cakes, or other high-calorie food made you feel fat?

18. Have you not gone out to social occasions (e.g., parties) because you have felt bad about your shape?

19. Have you felt excessively large and rounded?

20. Have you felt ashamed of your body?

21. Has worry about your shape made you diet?

22. Have you felt happiest about your shape when your stomach has been empty (e.g., in the morning)?

23. Have you thought that you are the shape you are because you lack self-control?

24. Have you worried about other people seeing rolls of flesh around your waist or stomach?

25. Have you felt that it is not fair that other women are thinner than you?

26. Have you vomited in order to feel thinner?

27. When in company have you worried about taking up too much room (e.g., sitting on the sofa or a bus seat)?

28. Have you been worried about your flesh being dimply?

29. Has seeing your reflection (e.g., in a mirror or shop window) made you feel bad about your shape?

30. Have you pinched areas of your body to see how much fat there is?

31. Have you avoided situations where people could see your body (e.g., communal changing rooms or swimming baths)?

32. Have you taken laxatives in order to feel thinner?

33. Have you been particularly self-conscious about your shape when in the company of other people?

34. Has worry about your shape made you feel you ought to exercise?

Supplementary S2. Body Appreciation Scale items

1. I RESPECT MY BODY

Never Seldom Some times Often Always

2. I FEEL GOOD ABOUT MY BODY

Never Seldom Some times Often Always

3. ON THE WHOLE, I AM SATISFIED WITH MY BODY

Never Seldom Some times Often Always

4. DESPITE ITS FLAWS, I ACCEPT MY BODY FOR WHAT IT IS

Never Seldom Some times Often Always

5. I FEEL THAT MY BODY HAS AT LEAST SOME GOOD QUALITIES

Never Seldom Some times Often Always

6. I TAKE A POSITIVE ATTITUDE TOWARD MY BODY

Never Seldom Some times Often Always

7. I AM ATTENTIVE TO MY BODY’S NEEDS

Never Seldom Some times Often Always

8. MY SELF-WORTH IS INDEPENDENT OF MY BODY SHAPE OR WEIGHT

Never Seldom Some times Often Always

9. I DO NOT FOCUS A LOT OF ENERGY BEING CONCERNED WITH MY BODY SHAPE OR WEIGHT

Never Seldom Some times Often Always

10. MY FEELINGS TOWARD MY BODY ARE POSITIVE, FOR THE MOST PART

Never Seldom Some times Often Always

11. I ENGAGE IN HEALTHY BEHAVIORS TO TAKE CARE OF MY BODY

Never Seldom Some times Often Always

12. I DO NOT ALLOW UNREALISTICALLY THIN IMAGES OF WOMEN PRESENTED IN THE MEDIA TO AFFECT MY ATTITUDES TOWARD MY BODY

Never Seldom Some times Often Always

13. DESPITE ITS IMPERFECTIONS, I STILL LIKE MY BODY

Never Seldom Some times Often Always
